# Too many journals? Towards a theory of repeated rejections and ultimate acceptance

**DOI:** 10.1007/s11192-015-1527-4

**Published:** 2015-01-22

**Authors:** Jan Oosterhaven

**Affiliations:** Faculty of Economics and Business, University of Groningen, P.O. Box 800, 9700 AV Groningen, The Netherlands

**Keywords:** Scientific journals, Rejection rates, Ultimate acceptance

## Abstract

Under a set of reasonable assumptions, it is shown that all manuscripts submitted to any journal will ultimately be published, either by the first journal or by one of the following journals to which a manuscript is resubmitted. This suggests that low quality manuscripts may also be published, which further suggests that there may be too many journals.

## Introduction

During a recent panel of scientific journal editors, all participating editors proudly announced that their journals maintained high quality standards.^1^ They all claimed that they only accepted the best articles for publication, as was “proven” by their high rejection rates that varied from as much as 70 to 85 %.^2^ Nevertheless, later on in the discussion, one of the editors complained that there were too many journals in their field and that almost all articles, including the ones of low quality, got published. Thus, the question arose which of the two statements is true and how the number of journals might influence the judgement.

## A base model with two generalizations

To analyse this question, we start with a base model of the cumulative acceptance or cumulative rejection of a single scientific manuscript, which is as simple as possible. Hence, in first instance, we do away with most complications that arise in reality, and introduce more realistic assumptions later on.

Assume a single journal, with a single editor, who has no memory, and a single manuscript that is accepted with a probability *a*, and rejected with a probability *r*, with no third option: thus *a* + *r* = 1. Assume further that this article, when rejected, is resubmitted endlessly, unless it is accepted. This last assumption obviously cannot be true, but reality comes close.^3^ With these assumptions, the probability of acceptance in the second round (i.e., after the first rejection) will be *r* *a*. In the third round it will be *r* ^2^ *a*. In the fourth round it will be *r* ^3^ *a*, and so on. The question then is whether the sum of this series converges to 1 or not, that is, whether the article ultimately will be published or not. The answer is that it will, as the *ultimate acceptance rate* equals

1 $$ u = a + r\,a + r^{2} a + r^{3} a + \cdots = \left( {1 + r + r^{2} + r^{3} + \cdots } \right)a = \left( {1{-}r} \right)^{ - 1} a = a^{ - 1} a = 1, $$

because r < 1.^4^ Note that the ultimate acceptance of this article does not depend on the rejection rate *r*, as long as that rejection rate does not increase with the number of resubmissions. This raises the side question, whether there is a type of increase in *r* that would prevent the ultimate acceptance of this article.

The only thing that does depend on *r* is the speed of convergence, i.e., after how many rounds the cumulative acceptance rate will reach which values. Table 1 gives the answer for a set of ordinary rejection rates of, respectively, 90, 80, 70, 60 and 50 %.^5^ With a rejection rate of only 50 % almost all articles, in fact 97 %, will be published in the 5th round, that is, after four resubmissions. But even with a rejection rate as high as 90 %, we still see that 41 % of all articles is published after 4 resubmissions.

**Table 1:** Cumulative acceptance of articles (in %) with the acceptance rates in the first row

1st round	10	20	30	40	50
2nd round	19	36	51	64	75
3rd round	27	49	66	78	88
4th round	34	59	76	87	94
5th round	41	67	83	92	97
10th round	65	89	97	99	100
15th round	79	96	99	100	100
20th round	88	98	100	100	100

Note that our wording has changed from a single article to a population of identical articles facing identical rejection rates in each (re)submission round. Obviously, this minor generalisation does not change (1), nor the conclusion. It does, however, raise the question whether our conclusion would change if we make our model much more realistic by allowing a multitude of journals.

Therefore, next, assume, without loss of generality, that we have *J* journals, instead of only one, and assume that each journal has its own rejection rate *r*_*j*_, rank-ordered in a column vector **r** or a diagonal matrix ${\hat{\mathbf{r}}}$ with **r** on its main diagonal. Again assume that there is no third option. Then the sum of each journal´s acceptance rate, $a_{j} \in {\mathbf{a}}$, and rejection rate, $r_{j} \in {\mathbf{r}}$, still equals one, $1 \in {\mathbf{i}}$, that is, **a** + **r** = **i**. Since, in this second model, there are more journals, there is no more need of the unrealistic assumption of a single editor without memory.

Instead, we assume that a manuscript that is rejected by journal *i* will be resubmitted to a different journal *j* with a transfer probability $p_{ij} \in {\mathbf{P}}$. The resubmission to a different journal, of course, implies that *p*_*ii*_ = 0. We, however, maintain the assumption that each rejected article is resubmitted, i.e., that authors do not withdraw their articles from the publication process. This implies that the matrix with the transfer probabilities has row sums that are equal to one, that is, ${\mathbf{P}}\,{\mathbf{i}} = {\mathbf{i}}$.

With these new, more realistic and more general assumptions, Eq. (1) changes into a matrix equation that defines the value of the *ultimate acceptance rate* of a manuscript that was originally submitted to journal *i*, as follows:

2 $$ u_{i} \in {\mathbf{u}} = \left[ {{\mathbf{I}} + \left( {{\hat{\mathbf{r}}}\,{\mathbf{P}}} \right) + \left( {{\hat{\mathbf{r}}}\,{\mathbf{P}}} \right)^{2} + \left( {{\hat{\mathbf{r}}}\,{\mathbf{P}}} \right)^{3} + \left( {{\hat{\mathbf{r}}}\,{\mathbf{P}}} \right)^{4} + \cdots } \right]\;{\mathbf{a}} = \left( {{\mathbf{I}} - {\hat{\mathbf{r}}}\,{\mathbf{P}}} \right)^{ - 1} {\mathbf{a}} $$

where ${\mathbf{I}} = {\hat{\mathbf{i}}}$, i.e., the unity matrix with ones on its main diagonal, and ()^−1^ = the inverse of the matrix (), for which holds that ()^−1^ () = **I**.^6^

The proof that all *u*_*i*_ in (2) are equal to one, as was the case with the single *u* in (1), only requires the assumption that rejected articles are not withdrawn from the publication process, i.e., that **Pi** = **i**, along with **a** + **r** = **i**:^7^

3 $$ {\mathbf{u}} = \left[ {{\mathbf{I}} + \left( {{\hat{\mathbf{r}}}\,{\mathbf{P}}} \right) + \left( {{\hat{\mathbf{r}}}\,{\mathbf{P}}} \right)^{2} + \cdots } \right]\;({\mathbf{i}} - {\mathbf{r}}) = {\mathbf{i}} + {\mathbf{r}} + {\hat{\mathbf{r}}}\,{\mathbf{Pr}} + \cdots - {\mathbf{r}} - {\hat{\mathbf{r}}}\,{\mathbf{Pr}} - \left( {{\hat{\mathbf{r}}}\,{\mathbf{P}}} \right)^{2} {\mathbf{r}} - \cdots = {\mathbf{i}} $$

Thus, also in this more general and more realistic case, all articles will still be published in the end, irrespective of the various rejection rates of the journals that they pass through. This is a rather strong statement.

The realism of this statement, of course, partly depends on the earlier side question whether there exist round-by-round increases in the rejection rates **r** that prevent the convergence of (2). The obvious cause for a higher rejection rate of the next journal in the series is that the content of the manuscript may become outdated, increasingly, either theoretically, methodologically, or empirically. The most important cause for a lower rejection rate, on the other hand, is that wise authors will use the referee reports of each earlier journal to improve its quality before each next submission.

Besides, in part to counteract the possible increase in the likelihood of a next rejection, authors usually react by choosing a new journal with a lower rejection rate, reflecting a lower status in the quality hierarchy of journals of which most authors are usually well aware. This implies that, besides **r**, also **P** will change with each resubmission round. Assume that authors strictly follow this strategy, then **P** becomes a triangular matrix, as we have rank-ordered **r** from journals with high rejections rates to journals with low rejection rates. With this assumption a more general version of (2) with varying **r** and **P** can still be solved relatively easily, as one journal drops out after each resubmission round. With these new assumptions, our third model for the *ultimate acceptance rates* becomes

4 $$ {\mathbf{u}} = {\mathbf{a}}_{1} + {\hat{\mathbf{r}}}_{1} {\mathbf{P}}_{1} \,{\mathbf{a}}_{2} + {\hat{\mathbf{r}}}_{1} {\mathbf{P}}_{1} \,{\hat{\mathbf{r}}}_{2} {\mathbf{P}}_{2} \,{\mathbf{a}}_{3} + {\hat{\mathbf{r}}}_{1} {\mathbf{P}}_{1} \,{\hat{\mathbf{r}}}_{2} {\mathbf{P}}_{2} \,{\hat{\mathbf{r}}}_{3} {\mathbf{P}}_{3} \,a_{4} + \cdots + {\hat{\mathbf{r}}}_{1} {\mathbf{P}}_{1} \,{\hat{\mathbf{r}}}_{2} {\mathbf{P}}_{2} \cdots {\hat{\mathbf{r}}}_{J - 1} {\mathbf{P}}_{J - 1} \,{\mathbf{a}}_{J} , $$

where the subscript indicates the number of the (re)submission round, and where the number of journals *J* determines the maximum number of (re)submission rounds.

The question now becomes whether **u** reaches unity before or after the maximum number of (re)submission rounds *J* is reached. The answer can no longer be given analytically, but needs to be based on empirical values of ${\hat{\mathbf{r}}}_{j}$ and **P**_*j*_.^8^ Nevertheless, it is clear that scientific areas with smaller numbers of journals will have a higher probability of having an ultimate acceptance rate smaller than 1, than areas with many competing journals.

## Conclusion and evaluation

In summary, we developed three, increasingly realistic theoretical models that all show that the dissenting editor of the panel, mentioned in the introduction, is right on both counts. High rejection rates may well go together with the ultimate acceptance of at least the majority of the initially submitted articles, while a large number of journals increases the probability of ultimate acceptance.

Adding an empirical foundation to our third and last theoretical model requires information on the rejection rates of journals and the resubmission behaviour of authors. The average rejection rates by journal are readily available, but how rejection rates change with resubmissions is difficult to establish, as editors mostly have no information about how many other journals, if any, rejected the article earlier, i.e., before it reaches their own desk. Further, information about the resubmission behaviour of authors is practically absent. A survey done via the editors of scientific journals probably leads to strategic and thus false answers.^9^ Interviewing authors directly, without the help of editors or publishers, may provide a way out.

Anyhow, a conclusive empirical proof of the validity of the above three models, will need time to develop, as the data needed for such a proof are not readily available. Still, Kochar (1986) found two studies that report ultimate acceptance rates of 85 % for manuscripts that were rejected by *The New England Journal of Medicine* and the *Journal of Clinical Investigation*, which have high own rejection rates of, respectively, 85 and 70 %.^10^

Our conclusion that most manuscripts will ultimately be accepted, therefore, not only has the theoretical backing given in this short article, but also has some initial empirical backing. This conclusion not necessarily, but most likely implies that too many manuscripts do get published in the end. The adjective “too” is justified because our conclusion implies that manuscripts of low quality also get published, unless one believes that the repeated refereeing process increases the quality of each manuscript sufficiently to warrant its ultimate publication.

## Footnotes

1 This panel was held during the 54th European Congress of the Regional Science Association International (RSAI) in Saint Petersburg, end of August 2014. The dissenting editor was Henk Folmer of *Letters in Spatial and Resource Sciences*, who, interestingly, despite his statement, was involved in setting-up yet another journal during the same congress.

2 There is indeed evidence that high rejection rates correlate with citation-based journal quality indicators (Yamazaki 1995; Pautasso and Schaefer 2010; Sugimoto et al. 2013).

3 Indirect evidence about the behaviour of authors after their manuscript is rejected is given by Altman and Baruch (2008). They received replies to a survey about their publishing strategies from 249 authors, and report a likelihood of only 1.11, respectively, 1.64, on a scale of 1–7, that authors “forget about a paper” after they get a minor, respectively, major revise & resubmit decision, whereas they report a likelihood of 6.77, respectively, 5.75 to resubmit to the original journal. Additional, more direct evidence follows from likelihoods around two, respectively, three to resubmit to a different journal, both with and without a rewrite. Together, this indicates that some manuscripts are withdrawn from publication after rejection, but also that this happens relatively seldom.

4 A search for the combination of “ultimate acceptance” and “manuscripts” with Google Scholar produced 1,090 hits, with 235 hits after 2010. Less than half of the latter hits were somehow related to our topic, but almost all of those dealt to the refereeing process of a single journal, which is not the topic of this article.

5 With 65 communication and journalism journals, the mean rejection rate reported was 81 %, with a standard deviation of 9 % (Stephen 2012).

6 The inverse of $\left( {{\mathbf{I}} - {\hat{\mathbf{r}}}\,{\mathbf{P}}} \right)$ exists because all row sums of ${\hat{\mathbf{r}}}\,{\mathbf{P}}$ are smaller than one (Nikaido 1970, p. 18).

7 An alternative proof of (3) starts with pre-multiplying $\left( {{\mathbf{I}} - {\hat{\mathbf{r}}}\,{\mathbf{P}}} \right)^{ - 1} \left( {{\mathbf{i}} - {\mathbf{r}}} \right) = i$ with $\left( {{\mathbf{I}} - {\hat{\mathbf{r}}}\,{\mathbf{P}}} \right)$.

8 When publications in online journals like PLOS ONE, which publishes over five thousand papers a year and has a rejection rate of about 30 % (Fein 2013), are also taken into account, (4) will reach unity much earlier, as **r** will decrease very quickly.

9 In fact, Altman and Baruch (2008, footnote 2), in an attempt to discover what authors do when they do not revise and resubmit (R&R) to the original journal, only found 17 out of 89 authors wiling to answer their survey; most likely because the survey was send via the editors of journals that did not receive an answer from these authors within 8 months after their R&R decision, such despite a strong promise of confidentiality.

10 Likewise, the editor of *Mass Communication & Society* (Perry 2008) has the impression that almost all manuscripts get published, either by the first, the second or the third journal to which they are submitted.
